# Isolating the speed factor is crucial in gait analysis for Parkinson’s disease

**DOI:** 10.3389/fnins.2023.1119390

**Published:** 2023-04-21

**Authors:** Aurélien Patoz, Davide Malatesta, Johannes Burtscher

**Affiliations:** ^1^Institute of Sport Sciences, University of Lausanne, Lausanne, Switzerland; ^2^Research and Development Department, Volodalen Swiss Sport Lab, Aigle, Switzerland

**Keywords:** unified Parkinson’s disease rating scale, stride analysis, kinematics, vertical ground reaction force, asymmetry, variability

## Abstract

**Introduction:**

Parkinson’s disease (PD) is characterized by an alteration of the walking gait, frequently including a slower self-selected walking speed (SSWS). Although the reduction of walking speed is inherent to people with PD, such speed reduction also represents a potential confounding factor that might partly explain the observed gait differences between PD and control participants.

**Methods:**

In this study, each participant walked along a 25 m level corridor during which vertical ground reaction force signals were recorded using shoes equipped with eight pressure sensors. Vertical ground reaction force signals (using statistical parametric mapping) and temporal and kinetic variables as well as their related variability and asymmetry (using Student’s *t*-test) were compared between PD (*n* = 54) and walking-speed-matched control subjects (*n* = 39).

**Results:**

Statistical parametric mapping did not yield significant differences between PD and control groups for the vertical ground reaction force signal along the walking stance phase. Stride time and single support time (equivalent to swing time) were shorter and peak vertical ground reaction force was larger in PD patients compared to controls (*p* ≤ 0.05). However, the single support time was no longer different between people with PD and healthy subjects when expressed relatively to stride time (*p* = 0.07). While single support, double support, and stance times were significantly more variable and asymmetric for PD than for the control group (*p* ≤ 0.05), stride time was similar (*p* ≥ 0.07).

**Discussion:**

These results indicate that at matched SSWS, PD patients adopt a higher cadence than control participants. Moreover, the temporal subdivision of the walking gait of people with PD is similar to healthy individuals but the coordination during the double support phase is different. Hence, this study indicates that isolating the speed factor is crucial in gait analysis for PD.

## Introduction

The prevalence of Parkinson’s disease (PD) is increasing faster than any other neurological disease (Dorsey and Bloem, 2018). Without disease-modifying treatments currently available and in view of heterogeneous disease manifestations and progression patterns complicating diagnosis, reliable molecular and behavioral biomarkers are urgently needed both to enable precision medicine and for recruitment of homogenous PD-subgroup populations for clinical trials.

Gait alterations in PD are well established (Mirelman et al., 2019) and can reduce quality of life and increase the risk of falling (Bloem et al., 2004; Boonstra et al., 2008; Creaby and Cole, 2018). Compared to age-matched control subjects, PD is frequently associated with a 0.17 m/s slower SSWS (Zanardi et al., 2021) and accompanied by a 0.16 m smaller stride length, 1.75 step/min higher cadence, 1.8% shorter swing time, and 1.8% longer double support time. In addition, people living with PD walk with a reduced range of hip, knee, and ankle motions (Carpinella et al., 2007; Dipaola et al., 2016; Monteiro et al., 2017), which causes the short stride length at SSWS (Dipaola et al., 2016; Monteiro et al., 2017).

Individuals with PD also present elevated stride time and swing time variability (del Olmo and Cudeiro, 2005; Frenkel-Toledo et al., 2005a; Alam et al., 2017), a pivotal and intrinsic difference to healthy people. Moreover, unilaterality of motor symptoms is a distinctive feature of PD (Hughes et al., 1992; Djaldetti et al., 2006) and causes postural instability and asymmetrical gait (Baltadjieva et al., 2006). Fling et al. (2018) observed larger swing duration and step time asymmetries and Arippa et al. (2022) reported larger asymmetries for step length and double support time in individuals suffering from PD compared to control participants.

Kinetic variables such as vertical ground reaction force data were also used to differentiate between PD and healthy individuals (Alam et al., 2017; Farashi, 2021) and to assess the severity of the disease (Veeraragavan et al., 2020; Balaji et al., 2021; Setiawan and Lin, 2021; Khera and Kumar, 2022). Although the vertical ground reaction force time-domain pattern during the stance phase was similar between PD and healthy individuals, its local characteristics (e.g., peak value) differed significantly (Farashi, 2021). The peak of vertical ground reaction force was larger and was observed later during the stance phase for patients with PD. This attests that they applied more force and needed more time for postural stabilization during the stance phase. Veeraragavan et al. (2020) assessed the usefulness of the vertical ground reaction force data to diagnose and evaluate early-stage PD. Such early-stage diagnosis would be urgently needed for selecting appropriate treatment strategies, improving quality of life, and eventually increasing the efficiency of future disease-modifying treatments (Mahlknecht et al., 2015).

These previous studies investigated kinematic and kinetic differences using summary metrics. Reduction to the summary-metric space, however, is not strictly necessary because statistical hypothesis testing can also be conducted in a continuous manner (Pataky, 2012). Indeed, one-dimensional biomechanical curves such as the ground reaction force signals are registrable and their fluctuations can be described and expressed as a function of the normalized stance duration (Cavanagh and Lafortune, 1980; Sadeghi et al., 2003). In this case, statistical analysis can be conducted on the original registered curves using statistical parametric mapping (SPM) (Friston et al., 2007). SPM was recently applied to investigate during which periods of the gait cycle significant differences of sagittal plane joint kinematics associated with PD occurred (Arippa et al., 2022). SPM allows direct visualization of where significant differences occur, which is a major advantage (Pataky, 2012).

The previously mentioned studies compared PD and healthy participants using SSWS, i.e., walking speed was not controlled. The reduction of walking speed in PD subjects compared to healthy individuals (Zanardi et al., 2021) was explained by a combination of reduced stride length and increased cadence (Carpinella et al., 2007; Monteiro et al., 2017). We suggest that this speed reduction might also represent a potential confounding factor that could partly explain the observed spatiotemporal, kinematic and kinetic differences between PD and control participants. Hence, the aim of the present study was to compare spatiotemporal and kinetic variables, their related variability and asymmetry between PD and walking-speed-matched control subjects. Comparison with speed-matched control individuals allowed us to investigate subjects walking at their SSWS, instead of a pre-imposed speed, permitting isolation of effects associated with walking speed. We hypothesized that (1) a shorter stride time and swing time, and longer double support time would still be observed for people with PD compared to healthy individuals when the walking speed is matched between groups, (2) PD would be associated with more variable and asymmetric walking gait, and (3) PD patients would have a larger peak vertical ground reaction force than healthy individuals. Furthermore, this study aimed to explore potential differences in the vertical ground reaction signal between people with PD and walking-speed-matched healthy individuals using SPM.

## Materials and methods

### Dataset description

The dataset used in this study was a publicly available gait dataset from Physionet (Goldberger et al., 2000) and contained recordings of the gait patterns of 93 PD patients and 73 control individuals. The PD patients continued to take antiparkinsonian medications according to their prescriptions at the time of experiment. These data were collected at the Movement Disorders Unit of the Tel-Aviv Sourasaky Medical center, Israel, in the frame of projects that were published in the following articles: Yogev et al. (2005), Hausdorff et al. (2007), and Frenkel-Toledo et al. (2005b). The study population was characterized with respect to sex, age, height, body mass, Hoehn-Yahr scale (Hoehn and Yahr, 1967), unified PD rating scale (Fahn et al., 1987), and timed up and go test (Mathias et al., 1986). All research activities were approved by Institutional Review Board (IRB; Human Studies Committee of Tel-Aviv Sourasky Medical Center) at the participating study site and participants provided written informed consent prior to entering the study.

### Experimental procedure

Participants performed a 2 min (Yogev et al., 2005; Frenkel-Toledo et al., 2005b) or 100 m (Hausdorff et al., 2007) free walking task. To remove any confounding effects of the walking speed on the gait variables, participants with similar walking speed were selected for the subsequent analysis. Based on the characteristics of the dataset (average SSWS of the entire dataset: 1.13 ± 0.22 m/s) and with the intent to obtain a sufficiently high number of participant data, participants walking on average at speeds between 1.0 and 1.3 m/s were included. This led to inclusion of 54 and 39 subjects in PD and control groups, respectively. Each participant walked along a 25 m level corridor using shoes equipped with eight pressure sensors (Ultraflex Computer Dyno Graphy, Infotronic Inc.), allowing to measure the vertical ground reaction force during walking. The gait signal was transferred to a recording unit (~1.5 kg) connected to the waist of the participant, digitized at 100 Hz, and stored on a memory card for analysis by a computer system.

### Data processing

The vertical ground reaction force signal corresponding to a single foot $\left(F_{z}\right)$ was obtained by summing the force values recorded by the eight sensors present under one shoe. $F_{z}$ signals were processed to remove noise by replacing any values less than 20 N by zero and were subsequently low-pass filtered at 15 Hz using a fourth-order Butterworth filter. Moreover, the total vertical ground reaction force signal $\left(F_{z,\mathrm{tot}}\right)$ was obtained by summing $F_{z}$ of each foot together.

To exclude startup effects, the first 5 s of data were discarded. Moreover, the turns made at both ends of the corridor during the free walking task were manually identified and removed for each participant so that only straight walking gait was compared between PD and control groups, leading to the analysis of 73 ± 14 strides per subject.

### Temporal variables

Foot-strike and toe-off events were detected separately for each foot by applying a 20 N threshold to $F_{z}$ . Stride and stance times were defined by the time between two consecutive foot-strike events of the same foot and between foot-strike and toe-off events of the same foot, respectively. Single and double support times were defined by the time between toe-off and foot-strike events of the contralateral foot and between foot-strike event of one foot and toe-off event of the contralateral foot, respectively. Stance time and single and double support times were also given as a percentage of stride time.

### Force related variables

First and second peak vertical force for each foot ( $F_{z,\mathrm{peak}\;1}$ and $F_{z,\mathrm{peak}\;2}$ ), total peak vertical force summing $F_{z}$ of each foot together $\left(F_{z,\mathrm{tot},\mathrm{peak}}\right)$ during stance, and the time at which these peaks occurred relative to the previous foot-strike event were extracted and given in ms and as a percentage of stride time. In addition, the dynamics around the gait weight transfer was characterized (Meurisse et al., 2016). To do so, the first local maximum of $F_{z}$ after the foot-strike event of the front foot $\left(F_{z,\mathrm{peak front}}\right)$ and the last local maximum of $F_{z}$ before the toe-off event of the back foot $\left(F_{z,\mathrm{peak back}}\right)$ were detected, and both the force difference $\left(\Delta F_{z}\right)$ and time duration (gait weight transfer; gwt) between these two maxima. Force variables were normalized by body weight (BW).

### Symmetry index

The symmetry index (SI) (Beck et al., 2018) was calculated for each temporal and force related variables as follows (Eq. 1):


(1) $$\mathrm{SI}=\frac{\left|X_{R}-X_{L}\right|}{0.5\left(\left|X_{R}\right|+\left|X_{L}\right|\right)}$$

where $X_{i}$ ( i=RorL) denotes either a temporal or a force related variable corresponding to the right (*R*) or left (*L*) foot. A perfectly symmetric gait corresponds to a SI of zero.

For all biomechanical measures, the values extracted from the free walking data collection for each participant were averaged for subsequent statistical analyses. Except for the symmetry indices, no distinction was made between left and right values, i.e., biomechanical measures were given as the average between their left and right values, because no information about asymmetric motor impairment was provided in the database. The variability of the temporal and force related variables was also quantified by computing the coefficient of variation, i.e., the ratio between standard deviation and mean values.

### Statistical analysis

Descriptive statistics are presented as mean ± standard deviation. Unpaired two-sided Student’s t-tests were used to compare participant characteristics, temporal and force related variables, and corresponding coefficient of variations and symmetry indices between PD and control groups. Differences in $F_{z}$ and $F_{z,\mathrm{tot}}$ (along the walking stance phase) between PD and control groups was examined using SPM and Student’s *t*-tests. To compare participants, the stance phase was normalized and therefore expressed in percentage. Statistical analysis was performed using spm1D (v0.4.6^1^) (Pataky, 2012), Python (v3.7.4^2^), and Jamovi (v1.6.23^3^) with a level of significance set at *p* ≤ 0.05.

## Results

Included PD patients had scores of 2.2 ± 0.3 according to the Hoehn-Yahr and 30 ± 10 according to the unified PD rating scale. The PD group required significantly more time in the timed up and go test than the control group (*p* ≤ 0.03; Table 1). Otherwise, PD and control groups were similar in terms of walking speed (selection criterium), age, height, and body mass (*p* ≥ 0.18; Table 1).

**Table 1:** Participant characteristics for Parkinson’s disease and control groups.

Characteristics	Parkinson’s disease	Control	*p*
Sex	*M* = 36; *F* = 18	*M* = 23; *F* = 16	NA
Age (year)	63 ± 9	65 ± 9	0.31
Height (cm)	167 ± 7	169 ± 9	0.38
Body mass (kg)	73 ± 11	74 ± 13	0.66
Walking speed (m/s)	1.15 ± 0.08	1.17 ± 0.09	0.18
Hoehn-Yahr scale	2.2 ± 0.3	0.0 ± 0.0	**<0.001**
Unified Parkinson’s disease rating scale	30 ± 10	0 ± 1	**<0.001**
Timed up and go test	10.8 ± 1.8	10.0 ± 1.4	**0.03**

Values are presented as mean ± standard deviation, M, male; F, female; and NA, not applicable. Significant differences (*p* ≤ 0.05) identified by Student’s *t*-tests are reported in bold.

Pearson’s correlation coefficient between the unified PD rating scale and walking speed for the 54 PD subjects was 0.36 and significant (*p* = 0.009).

The PD group had a significantly shorter stride time and single support time than the control group (*p* ≤ 0.04; Table 2). However, the single support time was no longer different between PD and control groups when expressed relatively to stride time (*p* = 0.07; Table 2). In addition, all temporal variables except stride time were significantly more variable for the PD than the control group, as shown by their larger coefficient of variation (*p* ≤ 0.05; Table 2). The same variables also indicate significantly less symmetry of the gait of PD patients as compared to control (*p* ≤ 0.005; Table 3).

**Table 2:** Temporal variables for Parkinson’s disease and control groups.

Temporal variables	Average		*p*	Coefficient of variation (%)		*p*
	Parkinson’s disease	Control		Parkinson’s disease	Control	
Stride time (ms)	1,071 ± 81	1,107 ± 80	**0.04**	2.2 ± 0.8	2.1 ± 0.6	0.37
Single support time (ms)	389 ± 35	409 ± 31	**0.007**	5.0 ± 2.1	3.6 ± 1.1	**<0.001**
Single support time (%)	36.3 ± 1.7	36.9 ± 1.3	0.07	4.7 ± 2.2	3.2 ± 1.2	**<0.001**
Double support time (ms)	146 ± 21	145 ± 19	0.72	10.3 ± 3.9	8.2 ± 2.7	**0.006**
Double support time (%)	13.7 ± 1.7	13.1 ± 1.3	0.07	9.6 ± 3.8	7.4 ± 2.5	**0.002**
Stance time (ms)	682 ± 54	698 ± 54	0.16	3.4 ± 1.3	2.9 ± 0.9	**0.05**
Stance time (%)	63.7 ± 1.7	63.1 ± 1.3	0.07	2.5 ± 1.2	1.8 ± 0.6	**<0.001**

Values are presented as mean ± standard deviation. Significant differences (*p* ≤ 0.05) identified by Student’s *t*-tests are reported in bold.

**Table 3:** Symmetry index of the temporal variables for Parkinson’s disease and control groups.

Symmetry index	Parkinson’s disease	Control	*p*
Stride time (%)	1.3 ± 0.4	1.2 ± 0.3	0.07
Single support time (%)	6.5 ± 4.2	4.1 ± 1.9	**0.001**
Double support time (%)	12.6 ± 6.9	8.6 ± 4.0	**0.002**
Stance time (%)	3.7 ± 2.4	2.5 ± 1.1	**0.005**

Values are presented as mean ± standard deviation. Significant differences (*p* ≤ 0.05) identified by Student’s *t*-tests are reported in bold.

$F_{z,\mathrm{tot},\mathrm{peak}}$ was significantly higher for the PD group than for the control group (*p* = 0.05; Table 4). In addition, $F_{z,\mathrm{peak}\;1}$ and $F_{z,\mathrm{peak front}}$ were significantly more variable (*p* ≤ 0.009; Table 4) and significantly less symmetric (*p* < 0.001; Table 5) for the PD than the control group.

**Table 4:** Force related variables for Parkinson’s disease and control groups.

Force related variables	Average		*p*	Coefficient of variation (%)		*p*
	Parkinson’s disease	Control		Parkinson’s disease	Control	
$F_{z,\mathrm{peak}\;1}$ (BW)	1.38 ± 0.21	1.34 ± 0.17	0.31	5.0 ± 2.9	3.6 ± 1.5	**0.009**
Time to $F_{z,\mathrm{peak}\;1}$ (ms)	163 ± 25	161 ± 23	0.62	12.9 ± 9.3	11.3 ± 7.1	0.37
Time to $F_{z,\mathrm{peak}\;1}$ (%)	15.0 ± 2.0	14.7 ± 2.1	0.54	12.3 ± 9.3	10.6 ± 7.2	0.33
$F_{z,\mathrm{peak}\;2}$ (BW)	1.24 ± 0.18	1.24 ± 0.16	0.93	5.4 ± 2.8	4.5 ± 2.2	0.11
Time to $F_{z,\mathrm{peak}\;2}$ (ms)	460 ± 57	464 ± 50	0.70	7.1 ± 3.1	7.6 ± 3.5	0.53
Time to $F_{z,\mathrm{peak}\;2}$ (%)	42.9 ± 3.8	41.9 ± 3.8	0.23	6.8 ± 3.3	7.0 ± 3.8	0.72
$F_{z,\mathrm{tot},\mathrm{peak}}$ (BW)	1.78 ± 0.23	1.69 ± 0.18	**0.05**	5.1 ± 2.2	4.8 ± 1.5	0.50
Time to $F_{z,\mathrm{tot},\mathrm{peak}}$ (ms)	63 ± 31	61 ± 16	0.70	26.4 ± 24.2	23.8 ± 28.5	0.64
Time to $F_{z,\mathrm{tot},\mathrm{peak}}$ (%)	5.9 ± 2.7	5.6 ± 1.6	0.47	26.0 ± 24.2	23.7 ± 28.9	0.67
$F_{z,\mathrm{peak front}}$ (BW)	1.35 ± 0.21	1.33 ± 0.17	0.59	7.0 ± 4.6	4.1 ± 2.2	**<0.001**
$F_{z,\mathrm{peak back}}$ (BW)	1.25 ± 0.17	1.24 ± 0.16	0.89	6.4 ± 3.7	5.4 ± 2.8	0.15
$\Delta F_{z}$ (BW)	0.11 ± 0.23	0.09 ± 0.21	0.70	49.2 ± 309.3	48.7 ± 94.0	0.99
Gait weight transfer (ms)	221 ± 67	242 ± 69	0.13	24.6 ± 11.2	21.0 ± 10.7	0.13

Values are presented as mean ± standard deviation. $F_{z,\mathrm{peak}\;1}$ and $F_{z,\mathrm{peak}\;2}$ , first and second peak vertical forces during stance; $F_{z,\mathrm{tot},\mathrm{peak}}$ , total peak vertical force during double support; $F_{z,\mathrm{peak front}}$ , first local maximum of the vertical force corresponding to a single foot after the foot-strike event of the front foot; $F_{z,\mathrm{peak back}}$ , last local maximum of the vertical force corresponding to a single foot before the toe-off event of the back foot; $\Delta F_{z}$ , difference between $F_{z,\mathrm{peak front}}$ and $F_{z,\mathrm{peak back}}$ . Significant differences (*p* ≤ 0.05) identified by Student’s *t*-tests are reported in bold.

**Table 5:** Symmetry index of the force related variables for Parkinson’s disease and control groups.

Symmetry index	Parkinson’s disease	Control	*P*
$F_{z,\mathrm{peak}\;1}$ (BW)	6.8 ± 3.8	4.3 ± 2.0	**<0.001**
Time to $F_{z,\mathrm{peak}\;1}$ (ms)	16.1 ± 16.7	12.3 ± 8.3	0.19
$F_{z,\mathrm{peak}\;2}$ (BW)	8.5 ± 5.7	6.8 ± 4.6	0.13
Time to $F_{z,\mathrm{peak}\;2}$ (ms)	9.3 ± 4.9	8.9 ± 5.3	0.67
$F_{z,\mathrm{tot},\mathrm{peak}}$ (BW)	8.2 ± 5.0	7.5 ± 3.4	0.48
Time to $F_{z,\mathrm{tot},\mathrm{peak}}$ (ms)	27.1 ± 29.3	19.1 ± 13.1	0.12
$F_{z,\mathrm{peak front}}$ (BW)	9.7 ± 6.9	4.7 ± 2.5	**<0.001**
$F_{z,\mathrm{peak back}}$ (BW)	9.2 ± 5.9	7.8 ± 5.1	0.23
$\Delta F_{z}$ (BW)	94.7 ± 57.4	85.1 ± 46.6	0.39
Gait weight transfer (ms)	28.6 ± 15.7	22.8 ± 13.7	0.07

Values are presented as mean ± standard deviation. $\;F_{z,\mathrm{peak}\;1}$ and $F_{z,\mathrm{peak}\;2},$ first and second peak vertical forces during stance; $F_{z,\mathrm{tot},\mathrm{peak}}$ , total peak vertical force during double support; $F_{z,\mathrm{peak front}}$ , first local maximum of the vertical force corresponding to a single foot after the foot-strike event of the front foot; $F_{z,\mathrm{peak back}}$ , last local maximum of the vertical force corresponding to a single foot before the toe-off event of the back foot; $\Delta F_{z}$ , difference between $F_{z,\mathrm{peak front}}$ and $F_{z,\mathrm{peak back}}$ . Significant differences (*p* ≤ 0.05) identified by Student’s *t*-tests are reported in bold.

**Figure 1:**
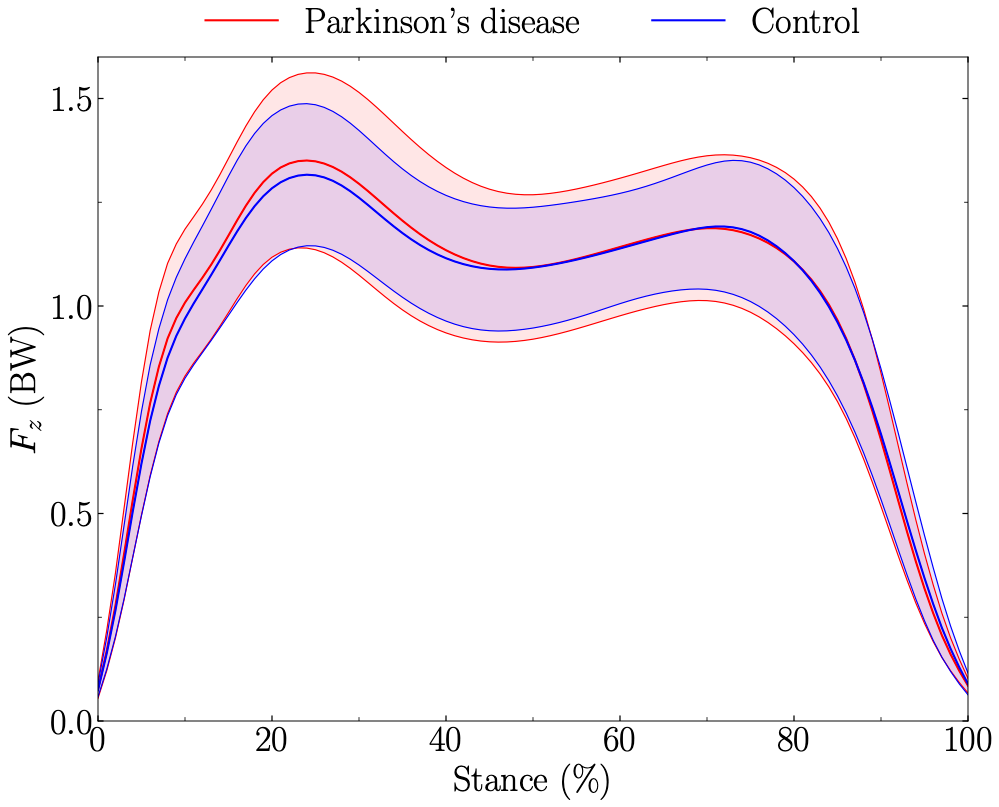
Vertical ground reaction force corresponding to a single foot (*F*_*z*_), expressed in body weight (BW), during stance (from foot-strike to toe-off of the same foot). Solid red and blue lines represent the average for Parkinson’s disease and control groups, respectively, while the standard deviations are given by the shaded areas. The *t*-test comparing Parkinson’s disease and control groups along the walking stance phase and obtained using statistical parametric mapping was not significantly different.

**Figure 2:**
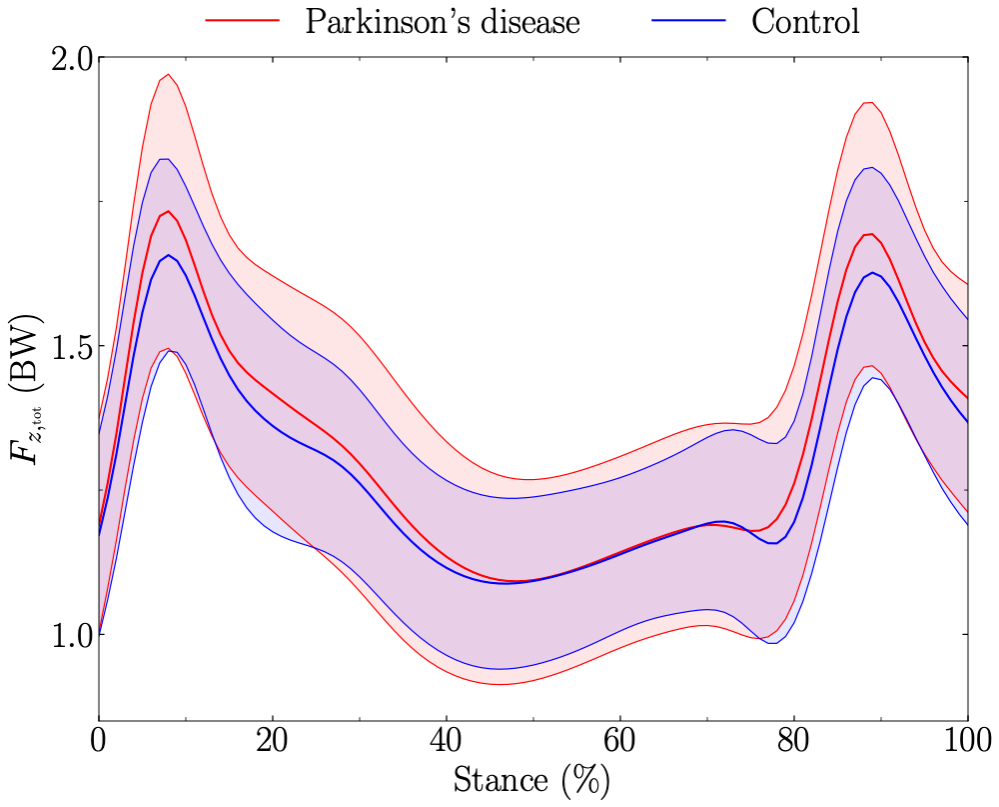
Total vertical ground reaction force (obtained by summing the vertical ground reaction force of each foot together; *F*_*z*,tot_), expressed in body weight (BW), during stance (from foot-strike to toe-off of the same foot). Solid red and blue lines represent the average for Parkinson’s disease and control groups, respectively, while the standard deviations are given by the shaded areas. The *t*-test comparing Parkinson’s disease and control groups along the walking stance phase and obtained using statistical parametric mapping was not significantly different.

Statistical parametric mapping did not reveal significant differences between PD and control groups for both $F_{z}$ and $F_{z,\mathrm{tot}}$ along the walking stance phase (Figures 1, 2).

## Discussion

Despite the growing understanding of the etiopathology of PD, accurate and especially early diagnosis of PD is still challenging (Rizzo et al., 2016). Beside the promising developments in tissue and fluid biomarkers, genetic analysis, and new diagnostic imaging and neurophysiology approaches, recent advances in the movement analysis field, including wearables and machine learning technologies, enable unprecedented deep phenotyping. These novel opportunities are expected to allow more accurate and objective diagnosis and disease progression prognosis that are key for early detection of PD symptoms and for the characterization of subtypes of PD. All of this is urgently needed for the selection of appropriate populations for clinical trials and for precision medicine.

Here we took advantage of a publicly available dataset on gait data of PD patients and controls and applied state of the art gait analysis. Our main objective was to evaluate the impact of temporal gait characteristics on other movement parameters. Although this possibility is usually not considered, differences in speed may confound other outcomes. To this end, we focused specifically on PD and control individuals with comparable SSWS.

According to the first hypothesis, a shorter stride time and single support time (equivalent to swing time) were observed for people with PD compared to healthy individuals, despite similar SSWS. However, double support time was not different in people with PD. Both double and single support times were also not different between PD and healthy subjects when expressed relatively to stride time. Supporting our second hypothesis, people with PD exhibited a more variable and asymmetric walking gait than healthy individuals. Moreover, a greater $F_{z,\mathrm{tot},\mathrm{peak}}$ (i.e., coordination index of double support phase) than healthy individuals characterized participants with PD, confirming our third hypothesis. Finally, no difference in the vertical ground reaction signal was observed between PD and walking-speed-matched healthy individuals, indicating that walking speed may indeed confound potentially distinguishing gait features of PD patients. At matched SSWS, PD patients adopted a higher cadence than control participants. While the temporal subdivision of walking gait was similar in PD and healthy individuals, differences in coordination during the double support phase underpin the notion that isolating the speed factor is crucial in gait analysis for PD.

The positive association (*r* = 0.36; *p* = 0.009) between disease severity assessed using the unified PD rating scale and walking speed reported herein seems counterintuitive and seemingly contradicts previous findings (Paker et al., 2015). A negative correlation between gait speed and disease severity would usually be assumed. This apparent contradiction is due to the very limited range of SSWS, due to our selection criteria of comparable SSWS. Whether this result indicates specific PD subgroups that are characterized by reduced SSWS loss, remains to be investigated.

In the present study we report a 36 ms (3.4%) significant shorter stride time for people with PD compared to healthy individuals (Table 2), corresponding to a 1.82 step/min higher cadence. This result corroborates the results of a recent meta-analysis, i.e., people with PD had a 1.75 step/min higher cadence than healthy subjects (Zanardi et al., 2021), extending these findings also for PD patients with similar SSWS. Indeed, removing the effect of the difference in SSWS between PD patients and healthy individuals emphasizes that the observed changes are likely and (directly) related to PD (and not slower SSWS). Hence, the isolation of the speed factor used in the present study further suggests that the usually slower SSWS of people with PD compared to healthy individuals (Zanardi et al., 2021) might mainly be due to a smaller stride length but not due to a higher cadence. Consequently, the generally slower SSWS of people with PD compared to healthy subjects might be caused by a smaller stride length that cannot be sufficiently compensated for by a higher cadence.

Patients with PD had a 20 ms (5.1%) significant shorter single support time than healthy individuals (Table 2). Nevertheless, both double and single support times were not different between people with PD and healthy subjects when expressed relatively to stride time (Table 2). These results suggest that the difference in single support time (and potential difference in double support time) was mainly due to the difference in stride time. Hence, PD and healthy individuals appear to have similar temporal subdivisions of their walking gait (double support time, single support time, and stance time) when expressed relatively to their stride duration.

The PD group had a 0.09BW (5.3%) significant greater $F_{z,\mathrm{tot},\mathrm{peak}}$ than the control group (Table 2), in line with previous findings (Farashi, 2021). However, SPM did not reveal differences in $F_{z,\mathrm{tot}}$ during stance between PD and control groups (Figure 2). This could partly be explained by the fact that $F_{z,\mathrm{tot},\mathrm{peak}}$ appears at different percentage of the stance among individuals, therefore partly smoothing out the difference when investigating $F_{z,\mathrm{tot}}$ during stance. Nonetheless, no difference in time to $F_{z,\mathrm{tot},\mathrm{peak}}$ (ms and %) was observed between PD and control groups (Table 4). Our results apparently contradict previous findings reporting $F_{z,\mathrm{tot},\mathrm{peak}}$ to generally appear later in PD patients compared to healthy individuals (Farashi, 2021), and suggest that this previous finding was likely also due to the difference in SSWS. There was no difference for $F_{z,\mathrm{peak}\;1}$ and $F_{z,\mathrm{peak}\;2}$ , and for the time at which these peaks occurred between PD patients and controls (Table 4). Therefore, these results indicate that at matched walking speed, postural stabilization is not necessarily prolonged for PD patients during the stance phase, as has been reported for non-speed controlled studies (Farashi, 2021). Noteworthy, even though the effect of walking speed on the time to peaks in the vertical force signal is known since decades (Nilsson and Thorstensson, 1989), this important aspect has not been sufficiently considered in PD-related gait analysis. Furthermore, the dynamics around the gait weight transfer (Meurisse et al., 2016) was similar between people with PD and healthy subjects (Table 4). Nevertheless, the significant difference in $F_{z,\mathrm{tot},\mathrm{peak}}$ suggests a difference in coordination during the double support phase, which needs further investigation.

Controlling speed effects further facilitates investigating differences in the pendulum-like mechanical energy transduction (Cavagna and Kaneko, 1977) and its repercussions on the energy cost of walking since both variables change as a function of walking speed (Malatesta et al., 2003; Peyré-Tartaruga et al., 2021). Hence, future studies should focus on concomitantly investigating the mechanics and energetics of walking in PD patients.

Temporal variables related to the stride (single support, double support, and stance times) were more variable and asymmetric in PD patients (Tables 2, 3), corroborating previous findings of higher swing time variability (Alam et al., 2017) and double support time asymmetry in PD (Arippa et al., 2022). Previously reported differences in stride time variability (Alam et al., 2017) were not observed in the present study (Tables 2, 3), possibly due to the selection criterium SSWS. Hence, our results suggest that although patients with PD and walking-speed-matched healthy individuals do not vary in terms of variability and asymmetry of their stride, differences within the subcomponents of their stride remain. According to the suggestion of Gabell and Nayak (1984), our patients with relatively low scores on the unified PD rating scale walking at SSWS may have an impaired balance control, related to an increased variability of double support time but with a maintained and normal automatic stepping mechanism. In fact, a failure of this latter mechanism may be associated with an increased stride time variability (Gabell and Nayak, 1984), characterizing walking of patients with higher PD severity (Hausdorff et al., 1998). Impaired balance control could be the first gait alteration in PD patients that may be followed by an altered automatic stepping mechanism corroborating previous findings showing that the degree of gait variability correlated with disease severity (Hausdorff et al., 1998). Future studies are needed to confirm this to enable the association of specific asymmetries and other gait abnormalities with distinct brain pathologies and PD subgroups.

The following limitations of the present study need to be considered. First, there might be a selection bias towards specific PD subpopulations especially due to the selection of patients with comparable SSWS that was also associated with relatively low scores on the used unified PD rating scale (30 ± 10). Second, people with PD continued to take antiparkinsonian medications according to their prescriptions at the time of experiment, which might likely decrease the potential differences compared to healthy individuals. Third, no kinematic analysis was performed, precluding the evaluation of the effect of matching the walking-speed on hip, knee, and ankle motions, hence warranting further investigations.

While we here only scratched the surface of trying to disentangle some gait factors that might be crucial for deep gait phenotyping of PD patients, the results of this study still demonstrate the huge potential of the increasingly powerful bioinformatics analysis of movement data for the characterization of PD patients. Our results further ask for the development of new testing protocols to detect subtle gait impairments in PD patients that potentially can be correlated to specific PD subgroups and the combination of such analyses with genetic, molecular, electrophysiological and brain-imaging biomarkers. The precise determination of gait modifications could ultimately facilitate precision medicine by identification and clustering of patient groups for subgroup-specific clinical trial allocation and intervention strategy selection (Zanardi et al., 2021).

## Conclusion

People with PD have a shorter stride time than walking-speed-matched healthy individuals but similar single support, double support, and stance times (expressed relatively to stride time). At matched SSWS, PD patients adopt a higher cadence than control participants, but people with PD and healthy individuals have a similar temporal subdivision of their walking gait. However, PD patients were characterized by a larger $F_{z,\mathrm{tot},\mathrm{peak}}$ than control group, suggesting a difference in coordination during the double support time. Moreover, people with PD exhibited a more variable and asymmetric walking gait than healthy individuals at similar SSWS. Taken together, this study demonstrates that isolating the speed factor is crucial in gait analysis for PD.

## Data availability statement

Publicly available datasets were analyzed in this study. This data can be found at: https://physionet.org/content/gaitpdb/1.0.0/.

## Ethics statement

The studies involving human participants were reviewed and approved by Institutional Review Board (IRB; Human Studies Committee of Tel-Aviv Sourasky Medical Center). The patients/participants provided their written informed consent to participate in this study.

## Author contributions

AP performed the data analysis and carried out the statistical analysis. AP and JB wrote the original draft of the manuscript. AP, JB, and DM critically revised the manuscript. All authors gave final approval for publication and agree to be held accountable for the work performed therein.

## Funding

This study was supported by the University of Lausanne (Switzerland). Open access funding by University of Lausanne.

## Conflict of interest

The authors declare that the research was conducted in the absence of any commercial or financial relationships that could be construed as a potential conflict of interest.

## Publisher’s note

All claims expressed in this article are solely those of the authors and do not necessarily represent those of their affiliated organizations, or those of the publisher, the editors and the reviewers. Any product that may be evaluated in this article, or claim that may be made by its manufacturer, is not guaranteed or endorsed by the publisher.

## Footnotes

^1^ https://spm1d.org

^2^ http://www.python.org

^3^ https://www.jamovi.org
